# Intelligent dementia friendly environment: A Preliminary Study on the Feasibility of Integrating Assistive Humanoid Robots, Wearable Sensors, and Spatial Technology for maintaining quality of life

**DOI:** 10.1002/alz.094203

**Published:** 2025-01-09

**Authors:** Arshia A Khan, Rupak Kumar Das, Rana Z Imtiaz

**Affiliations:** ^1^ University of Minnesota Duluth, Duluth, MN USA; ^2^ Pennsylvania State University, State College, PA USA; ^3^ Kentucky College of Osteopathic Medicine, PikeVille, KY USA

## Abstract

**Background:**

Integrating humanoid robots, wearable sensors, and spatial technology into an intelligent dementia‐friendly living space is crucial for tailoring personalized and supportive environments, thereby addressing the unique needs of individuals affected by dementia and maintaining quality of life.[1‐10].

**Methods:**

We programmed Pepper, a humanoid robot, for independent verbal communication to interact, tell jokes, and offer medications. To validate our assistive robot integrated with a sensor‐based system, we built a specifically designed living space optimized for sensor functionality and conducted a 90‐minute study involving 32 healthy participants. The participants performed various activities after consenting, filling pre and post‐experiment surveys and pre, during, and post‐sensor readings. The activities comprised of administration of a placebo sugar pill by the robot, treadmill exercises following the Bruce submaximal treadmill protocol, engagement in cognitively stimulating activities, wandering detection using spatial sensors, media presentation to induce higher arousal state, performance of some daily tasks such as preparing short meals, folding towels, cleaning the floor, and an interaction with the robot. These activities aimed to assess sensor functionality, simulate real‐world scenarios, and monitor physiological responses during various tasks, contributing to the evaluation of the integrated sensor system. Various physiological data, including Electrodermal Activity (EDA), blood pressure, and Electrocardiogram (EKG), were gathered using distinct wearable sensors throughout the experiment. To gauge the subjective experiences of participants, two surveys were employed: the Presentation survey, conducted during the International Affective Digital Sound (IADS) presentation, and the Post‐Study survey, distributed on paper after the study’s completion.

**Results:**

By incorporating pressure sensors in chairs and the robot’s emotion detection system, participants' moods were accurately assessed, aligning with self‐reported moods in 95% of cases. Furthermore, door sensors consistently identified exiting behavior, and wall‐mounted PIR sensors successfully recognized wandering behavior. Wearable sensors, such as EDA and blood pressure sensors, in conjunction with robot interactions, detected physiological changes during diverse activities, with the most notable impact observed during interactions with the robot. Conclusion The findings suggest that an intelligent living space specifically designed for dementia care can effectively preserve the quality of life for affected individuals, particularly through robot interactions, showcasing promising effectiveness.

